# Increased risk of admission to neonatal intensive care unit in neonates born to mothers with pregestational diabetes

**DOI:** 10.1007/s00431-025-06170-0

**Published:** 2025-05-22

**Authors:** Dearbhla Hillick, Daniel O’Reilly, Lauren Murphy, Fionnuala Breathnach, Naomi McCallion

**Affiliations:** 1 Department of Neonatology, Rotunda Hospital, Dublin, Ireland; 2 Department of Paediatrics, Royal College of Surgeons in Ireland, Dublin, Ireland; 3 Department of Obstetrics and Gynaecology, Rotunda Hospital, Dublin, Ireland; 4 Department of Obstetrics and Gynaecology, Royal College of Surgeons in Ireland, Dublin, Ireland

**Keywords:** Gestational diabetes, Hypoglycaemia, Neonatal intensive care unit, Type 1 diabetes, Type 2 diabetes

## Abstract

The purpose of this study was to describe how maternal diabetes impacts admission to the neonatal intensive care unit to support healthcare professionals when counselling patients. The primary outcome was admission rates. A retrospective observational cohort study of 25,238 births was conducted at an Irish tertiary maternity hospital from January 2018 to December 2020. Cases of pregestational and gestational diabetes were examined for neonatal intensive care admission outcomes. R statistical analysis software was used. There were 3905 live neonates born between 34 and 42 weeks to mothers with diabetes (*N* = 67 type 1 diabetes, *N* = 60 type 2 diabetes, *N* = 3712 gestational diabetes, *N* = 5 mature onset diabetes, excluded *N* = 61). There was a statistically significant difference in mean gestational age: 37 + 1 (weeks/days) (95% CI 36 + 6–37 + 4), 38 + 1 (95% CI 37 + 5–38 + 3, *p* = 0.0019), and 39 (95% CI 38 + 6–39 + 1, *p* ≤ 0.001) in type 1, type 2, and gestational diabetes cohorts. Admission rate was 13.4% with significant differences between the subgroups: 41.8% [95% CI 2.33–4.58, RR 3.32], 31.1% [95% CI 1.55–3.50, RR 3.89], and 12.5% [95% CI 0.12–0.14, RR 0.133] in type 1, type 2, and gestational diabetes cohorts. A higher percentage of mothers with pregestational diabetes (42.9% and 31.5%) were discharged before their infants, versus 21.2% of gestational diabetes.

*Conclusion*: Neonates of mothers with pregestational diabetes have a significantly higher admission rate. The type 1 diabetes cohort were born earlier and had higher birth weight centiles. Hypoglycaemia remains a significant risk for all subgroups.

**Table:** 

**What is known:** *• Infants of mothers with T1DM are born earlier and have higher morbidity rates compared to infants of mothers with T2DM or GDM.*
**What is new:** *• A higher proportion of neonates born to T2DM mothers were admitted due to severe/refractory hypoglycemia, however hypoglycemia was detected after admission in many neonates transferred to NICU for other reasons. It remains a significant risk and requires vigilance of all neonates born to mothers with pregestational or gestational diabetes.* *• Mothers with pre-gestational diabetes were more likely to be discharged home while their infant remains in NICU.*

**Supplementary Information:**

The online version contains supplementary material available at 10.1007/s00431-025-06170-0.

## Introduction

The prevalence of diabetes in pregnancy has increased in recent years, both for pregestational (type 1 and type 2 diabetes) [1] and gestational diabetes (GDM) [2]. The increasing incidence of pregestational diabetes diagnoses post-partum may complicate differentiation. Some individuals initially identified during screening with GDM may be subsequently diagnosed with type 2 diabetes (T2DM) post-partum [3] [4], indicating possible undiagnosed pregestational diabetes. Universal screening at the first antenatal appointment is now recommended to identify unrecognised diabetes in pregnancy [3]. Early diagnosis and strict glucose control in the antenatal period have been shown to significantly reduce maternal and neonatal complications and improve outcomes [5]. Neonates of mothers with pregestational diabetes and time in range target < 70% are more likely to be admitted to the neonatal intensive care unit (NICU), given intravenous glucose, and have respiratory distress syndrome and a longer hospital stay [6]. An Irish retrospective cohort study found that almost half of neonates born to mothers with pregestational diabetes were admitted to NICU [7]. Reported NICU admission rates for neonates of mothers with GDM are extremely variable, ranging from 10 to 30% [8, 9]. The estimated length of newborn hospital stay has been reported as almost double that observed in non-diabetes pregnancy [9] and significantly shorter hospital stay is reported in neonates of mothers with GDM than pregestational [10]. Common causes of admission include neonatal hypoglycaemia, perinatal acidosis, and transient respiratory morbidity. For term and near-term neonates, maternal insulin-dependent diabetes has been shown to be an independent risk factor for respiratory morbidity [11]. It is important to counsel mothers on risks and expectations for the newborn period [12, 13].

The aim of our study is to describe how the type of maternal diabetes impacts admission to NICU and to provide up-to-date, local data to support healthcare professionals when counselling patients with diabetes in pregnancy. We hypothesised that neonates born to mothers with type 1 diabetes (T1DM) would have a higher NICU admission rate and a longer length of stay than their T2DM and GDM counterparts.

## Methods

**Fig. 1:**
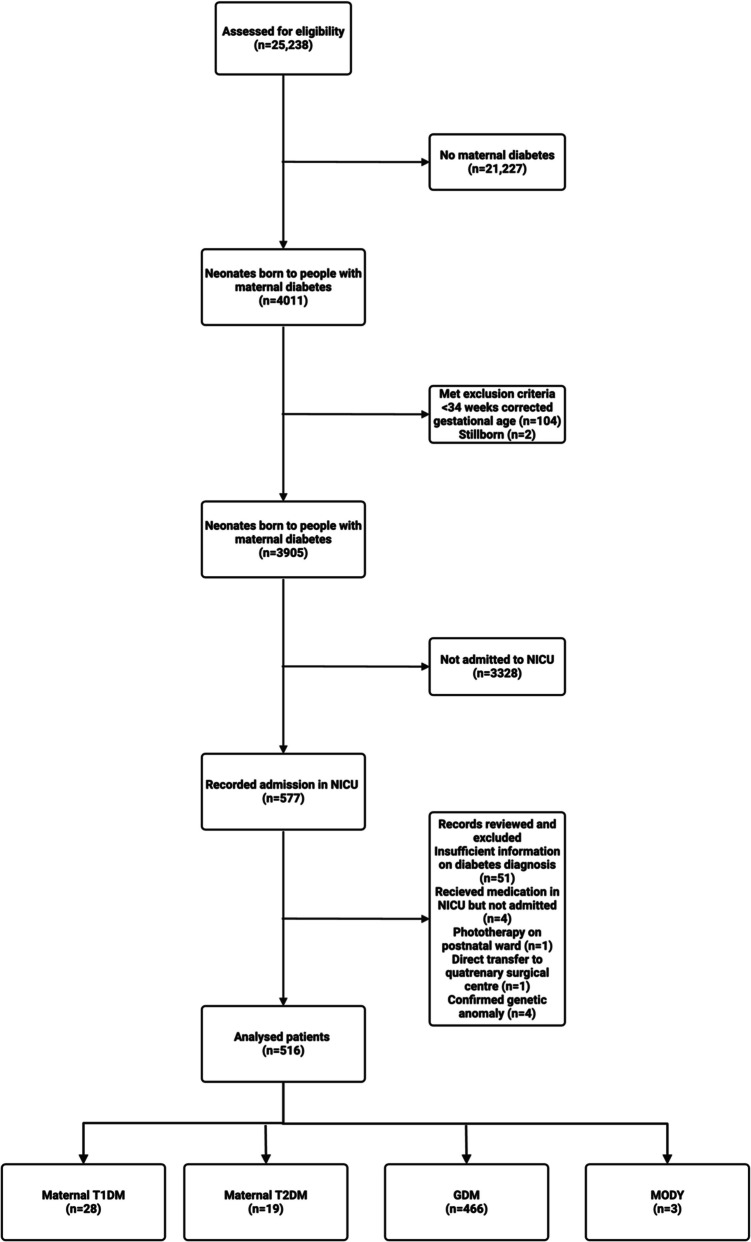
Consort diagram showing the flow of participants through each stage of our retrospective cohort study

A retrospective observational cohort study of 25,238 consecutive births was conducted at an Irish tertiary maternity hospital from January 2018 to December 2020 inclusive. We identified 3905 neonates born to mothers with pregestational and gestational diabetes between 34 + 0 and 42 + 0 (weeks/days) gestation via the Hospital In-Patient Enquiry (HIPE) computer system (see Fig. 1). Singletons and multiples were included. Neonates born less than 34 weeks were excluded as this degree of prematurity and its associated complications was likely to drive admission rate and clinical outcome rather than the type of maternal diabetes. Mothers with diabetes were identified by their type of diabetes through HIPE. Women with risk factors for GDM including obesity, polycystic ovarian syndrome, large-for-gestational-age fetus, south Asian, African or middle Eastern ethnicity, previous personal history of GDM, or family history of diabetes are offered an oral glucose tolerance test between 24 + 0 and 28 + 0 weeks in our hospital. A positive test confirms a diagnosis of GDM.

Gestational age, birth weight, mode of delivery, maternal age, and type of diabetes were collected through HIPE. Electronic patient records of NICU-admitted neonates were then reviewed to identify further admission details and maternal characteristics.

The primary outcome was admission to the NICU. Secondary outcomes included:

- Neonatal demographics and maternal obstetric features: gender, gestational age at birth, birth weight, mode of delivery, induction of labour, presence of meconium, pathological CTG, and APGAR scores at 5 min.
- Details of the NICU admission: age of the infant, primary indication and length of stay, need for respiratory or intravenous fluid support, and presence of any hypoglycaemia (defined as a blood glucose level < 3.0 mmol/l using ACCU-CHEK inform II system® manufactured by ROCHE diagnostics) during the admission stay. Our hospital aims for early feeding within the first hour, and glucose screening commences within the first 3 to 4 h of life, prior to the second feed [14]. The term respiratory distress was used to collate all infants admitted with clinical signs of respiratory distress, regardless of underlying pathophysiology.
- The admission outcome is defined as discharge to home, transfer to another hospital, or death.
- Maternal characteristics in the NICU-admitted cohort were collected as potential confounders: age, smoking status, body mass index (BMI) > 30 kg/m^2^, pre-eclampsia or pregnancy-induced hypertension, history of perinatal death in a prior pregnancy, use of antenatal steroids, assisted reproduction, and type and treatment of diabetes during pregnancy.

Research approval was obtained from the Rotunda Hospital Research Advisory Group. Data was irrevocably anonymised at collection; therefore, informed consent was not obtained. Research was conducted in accordance with the Declaration of Helsinki and European General Data Protection Regulation.

Statistical analysis was performed using R statistical analysis software [15]. To examine how diabetes sub-type affected admission and preterm birth, each diagnosis was fitted within a quasi-Poisson regression, with risk ratios generated through exponentiating the generated coefficients. Prematurity and birth weight were compared across groups using a two-tailed ANOVA, with Tukey’s honest significant difference test applied post hoc to establish the exact nature of the differences described. Comparisons between categorical variables (diabetes diagnosis versus indication for NICU admission) were performed using a chi-squared test. Comparison of centiles was performed using a permutation test, using the package “rcompanion” [16]. Graphs of data were generated on R using a variety of packages [17, 18].

## Results

Diabetes complicated 15.6% of pregnancies ≥ 34 weeks, see Fig. 1 for the flow of study participants.

**Fig. 2:**
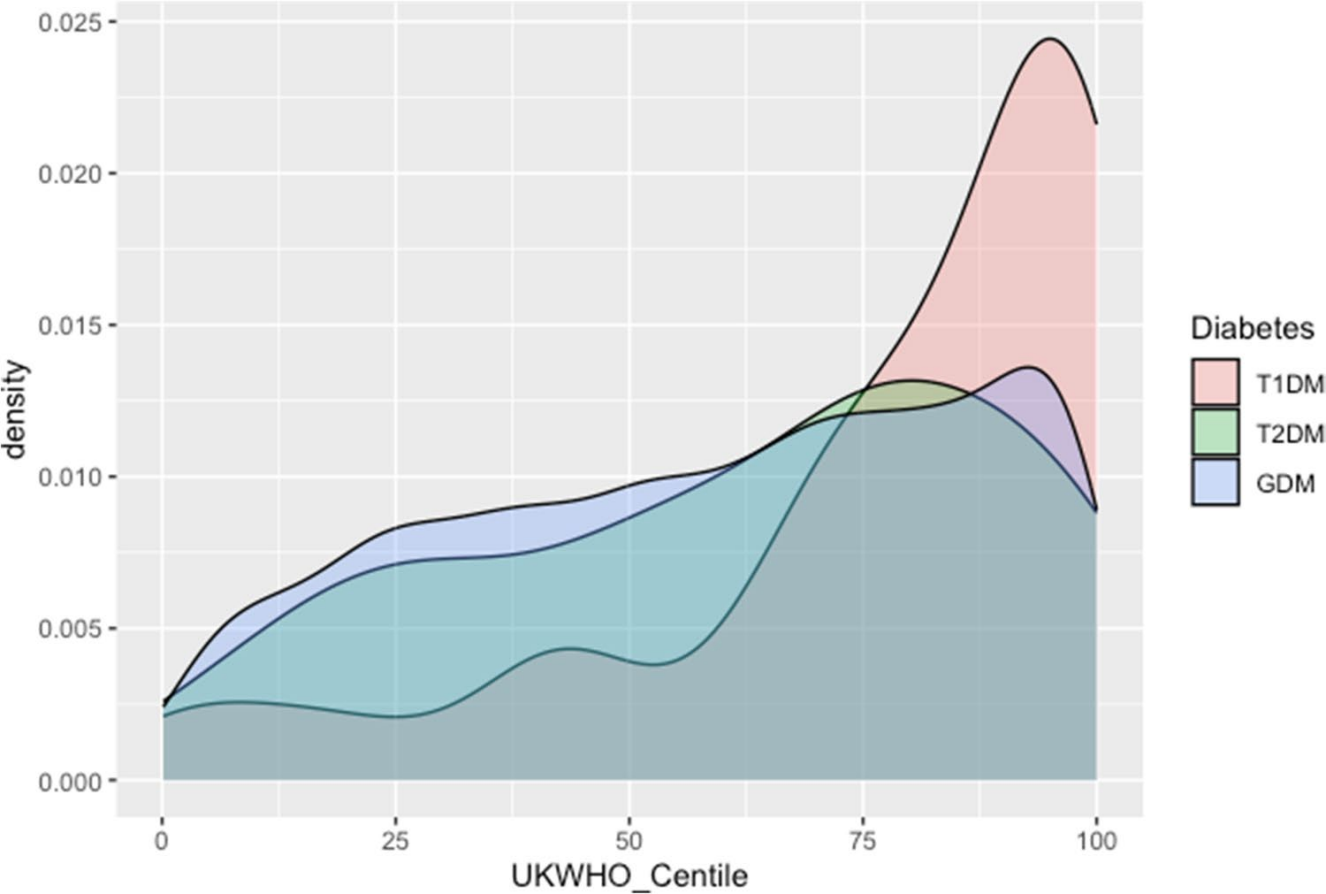
Density plot comparing birth weight centiles of three cohorts of neonates with maternal diabetes. T1DM, type 1 diabetes mellitus; T2DM, type 2 diabetes mellitus; GDM, gestational diabetes. The T1DM cohort is skewed heavily to higher centiles versus their T2DM and GDM counterparts

Patient demographic data are presented in Table 1, and maternal obstetric features are outlined in the supplemental material (appendix 2). There was a statistically significant difference in mean gestational age at delivery, with neonates of mothers with T1DM born significantly earlier at 37 + 1 (95% CI 36 + 6–37 + 4) versus 38 + 1 (95% CI 37 + 5–38 + 3, *p* = 0.0019) and 39 + 0 (95% CI 38 + 6–39 + 1, *p* ≤ 0.001) in the T2DM and GDM cohorts (appendix 1). Despite the difference in gestational age at delivery, there was no statistical difference in birth weight (*p* = 0.49). Using UK-WHO centiles, T1DM neonates were statistically larger at the 25 th (T1DM vs T2DM *p* = 0.0042, T1DM vs GDM *p* ≤ 0.001, T2DM versus GDM *p* = 0.57), median (T1DM vs T2DM *p* ≤ 0.0001, T1DM vs GDM *p* ≤ 0.0001, T2DM versus GDM *p* = 0.28), and 75 th centiles (T1DM vs T2DM *p* ≤ 0.0001, T1DM vs GDM *p* = 0.0009, T2DM vs GDM *p* = 0.955), as demonstrated in Fig. 2. The median APGAR score at 5 min was similar across all three cohorts. A higher proportion of these neonates were born via caesarean Sect. (74.6%) than those in the T2DM (52.5%) and GDM 43.8%) cohorts (appendix 2). Characteristics of neonates who did not require admission to the NICU are described in Table 4.

**Table 1:** Demographics of neonates born to mothers with pre- and gestational diabetes who were admitted to NICU

Neonatal characteristics	Neonates born to mothers with T1DM (*n* = 28)	Neonates born to mothers with T2DM (*n* = 19)	Neonates born to mothers with GDM (*n* = 466)	Neonates born to mothers with MODY (*n* = 3)	All neonates born to mothers with pre- and gestational diabetes (*n* = 516)
Gender (male/female)	12/16	10/9	262/204	0/3	284/232
Gestational age at birth (Median (IQR))	36 + 3 (35 + 4–37 + 3)	37 + 6 (36 + 6–38 + 1)	38 + 2 (37 + 0–39 + 3)	38 + 4 (38 + 1–39 + 1)	38 + 1 (36 + 6–39 + 2)
Birth weight (median and IQR)	3350 (2863–3793)	3540 (2995–3825)	3265 (2753–3700)	3390 (3070–3390)	3280 (2760–3730)
Apgar at 5 min (Median (IQR))	9 (8–10)	10 (10–10)	10 (9–10)	10 (9–10)	10 (9–10)

*T1DM*, type 1 diabetes mellitus; *T2DM*, type 2 diabetes mellitus; *GDM*, gestational diabetes mellitus; *MODY*, mature onset diabetes; *IQR*, interquartile range

The admission rate to NICU differed significantly between the subgroups (Table 2): 41.8% [95% CI 2.33–4.58, RR 3.32] versus 31.1% [95% CI 1.55–3.50, RR 3.89] and 12.5% [95% CI 0.12–0.14, RR 0.133] in the T1DM, T2DM, and GDM cohorts, respectively. Our background hospital NICU admission rate for neonates ≥ 34 weeks is 11.5%.

**Table 2:** Outcomes of neonates born to mothers with pre- and gestational diabetes who were admitted to NICU

	Neonates born to mothers with T1DM (*n* = 28)	Neonates born to mothers with T2DM (*n* = 19)	Neonates born to mothers with GDM (*n* = 466)	Neonates born to mothers with MODY (*n* = 3)	All neonates born to mothers with pre- and gestational diabetes (*n* = 516)
**Admission rate** **(%, RR)**	28/67 (41.8%, 3.32)	19/61 (31.1%, 3.89)	466/3715 (12.5%, 0.133)	3/5 (60%)	516/3844 (13.4%)
**Age of admission (hours)** **(Median (IQR))**	1 (0–3.75)	4 (1–12.5)	3 (0–24)	2 (2–2)	3 (0–22)
** > 1 admission**	0/28 (0%)	2/19 (10.5%)	21/466 (4.5%)	0/3 (0%)	23/516 (4.5%)
**Admitted from home**	1/28 (3.6%)	2/19 (10.5%)	46/466 (9.9%)	0/3 (0%)	49/516 (9.5%)
**Intravenous fluids**	26/28 (92.9%)	16/19 (84.2%)	314/466 (67.4%)	3/3 (100%)	359/516 (69.6%)
**Respiratory support**					
**• No support**	15/28 (53.6%)	16/19 (84.2%)	295/466 (63.3%)	3/3 (100%)	329/516 (63.8%)
**• Oxygen**	4/28 (14.3%)	2/19 (10.5%)	88/466 (18.9%)	0/3 (0%)	94/516 (18.2%)
**• NIV**	7/28 (25%)	1/19 (5.3%)	45/466 (9.7%)	0/3 (0%)	53/516 (10.2%)
• Intubation*	2/28 (7.1%)	0/19 (0%)	38/466 (8.2%)	0/3 (0%)	41/516 (7.9%)
**Hypoglycaemia during admission**†	25/28 (89.3%)	14/19 (73.7%)	227/466 (48.7%)	3/3 (100%)	266/516 (51.6%)
**Length of stay (days)** **(Median (IQR))**	3.5 (2–7.15)	3.1 (1.3–8.35)	2 (1.1–3.8)	3.8 (0.9–4.1)	2 (1.1–4)
**Maternal discharge before neonate**	12/28 (42.9%)	6/19 (31.5%)	99/466 (21.2%)	0/3 (0%)	119/516 (23.1%)
**Discharge outcome**					
**• Discharged home**	26/28 (92.9%)	19/19 (100%)	438/466 (94%)	2/3 (66%)	485/516 (94%)
**• Transferred to another hospital**	2/28 (7.1%)	0/19 (0%)	27/466 (5.8%)	1/3 (33.3%)	30/516 (5.8%)
**• Death**	0/28 (0%)	0/19 (0%)	1/466 (0.2%)	0/3 (0%)	1/516 (0.2%)
**Cause of death**			HIE with redirection — 1		

^*^Intubation for ventilation or INSURE

^†^Hypoglycaemia is defined as a blood glucose level < 3.0 mmol/l using the ACCU-CHEK inform II system® manufactured by ROCHE diagnostics

*T1DM*, type 1 diabetes mellitus; *T2DM*, type 2 diabetes mellitus; *GDM*, gestational diabetes mellitus; *MODY*, mature onset diabetes; *RR*, relative risk; *IQR*, interquartile range; *NIV*, non-invasive ventilation; *CDH*, congenital diaphragmatic hernia

Indications for admission to NICU are outlined in Table 3. The two most common indications for admission in T1DM and GDM cohorts were early respiratory distress, followed by severe/refractory hypoglycaemia, whereas the T2DM group was most likely to be admitted for severe/refractory hypoglycaemia at a later stage and had fewer respiratory problems. Our hospital defines severe hypoglycaemia as a blood glucose level < 1.8 mmol/l. Refractory hypoglycaemia is defined as > 2 episodes of a blood glucose < 2.6 mmol/l despite supplemental formula or oral glucose 40% gel. Hypoxic ischaemic encephalopathy (HIE) was uncommon across all groups, and admissions for perinatal acidosis were observed only in the GDM cohort. Correlation plots of reason for admission and percentage contribution of diagnosis are outlined in the supplemental material (appendix 3 and 4).

**Table 3:** Clinical indications for admission to NICU for neonates born to mothers with diabetes

Indication for admission	Neonates born to mothers with T1DM (*n* = 28)	Neonates born to mothers with T2DM (*n* = 19)	Neonates born to mothers with GDM (*n* = 466)	Neonates born to mothers with MODY (*n* = 3)	All neonates born to mothers with pre- and gestational diabetes (*n* = 516)
Respiratory distress	17/28 (60.7%)	4/19 (21%)	178/466 (38.2%)	0/3 (0%)	199/516 (38.6%)
Severe or refractory hypoglycaemia	9/28 (32.1%)	9/19 (47.4%)	65/466 (13.9%)	3/3 (100%)	86/516 (17.2%)
Jaundice	0/28 (0%)	3/19 (15.8%)	65/466 (13.9%)	0/3 (0%)	68/516 (13.2%)
Suspected congenital anomaly	0/28 (0%)	0/19 (0%)	25/466 (5.4%)	0/3 (0%)	25/516 (4.8%)
Feeding problems	0/28 (0%)	1/19 (5.3%)	25/466 (5.4%)	0/3 (0%)	26/516 (5%)
IUGR/LBW	0/28 (0%)	1/19 (5.3%)	24/466 (5.2%)	0/3 (0%)	25/516 (4.8%)
Suspected sepsis	0/28 (0%)	0/19 (0%)	24/466 (5.2%)	0/3 (0%)	24/520 (4.7%)
Perinatal acidaemia	0/28 (0%)	0/19 (0%)	22/466 (4.7%)	0/3 (0%)	22/516 (4.3%)
HIE	1/28 (3.6%)	0/19 (0%)	4/466 (0.9%)	0/3 (0%)	5/516 (1%)
Polycythaemia	0/28 (0%)	0/19 (0%)	4/466 (0.9%)	0/3 (0%)	4/516 (0.8%)
None of the above	1/28 (3.6%)	1/19 (5.3%)	30/469 (6.4%)	0/3 (0%)	32/516 (6.2%)

*T1DM*, type 1 diabetes mellitus; *T2DM*, type 2 diabetes mellitus; *GDM*, gestational diabetes mellitus; *MODY*, mature onset diabetes; *HIE*, hypoxic ischaemic encephalopathy; *IUGR*, intrauterine growth restriction; *LBW*, low birth weight

Secondary neonatal outcomes are described in Table 2. The T1DM cohort were admitted earlier and had a longer median length of stay than the T2DM and GDM cohorts. A higher proportion of the T1DM cohort required respiratory support and experienced neonatal hypoglycaemia during their admission. A similar rate of discharge to home was seen across the three largest cohorts (92.9% vs 100% vs 94%) although a higher proportion of T1DM and T2DM mothers were discharged home before their infant (42.9% and 31.5% versus 21.2%). Transfers to quaternary hospitals for surgical management or repatriation to local hospitals accounted for 5.8% of all neonates. One neonate died of HIE (Table 3). Characteristics of those who did not require NICU admission are described in Table 4.

**Table 4:** Characteristics of neonates born to mothers with diabetes over 34 weeks who did not require admission to NICU

	Non-admitted neonates > 34 weeks born to people with a diabetes diagnosis in pregnancy
Total	3328
Male (*n*, %)	1716/3328 (51.6%)
Gestational age (mean)	39 + 1
Birth weight in grams (mean)	3487
Recorded maternal diabetes diagnosis	T1DM (*n* = 39/3328, 1.2%) T2DM (*n* = 41/3328, 1.2%) GDM/not specified (3246/3328, 97.5%) MODY (*n* = 2/3328, < 0.01%)

Maternal characteristics of neonates admitted to the NICU are demonstrated in Table 5.

**Table 5:** Maternal characteristics of infants requiring admission to the NICU

Maternal characteristics	Mothers with T1DM (*n* = 28)	Mothers with T2DM (*n* = 19)	Mothers with GDM (*n* = 466)	Mothers with MODY (*n* = 3)	All mothers of admitted infants (*n* = 520)
**Age (years)** **(Median (IQR))**	35 (31–40.8)	34 (30.5–40.5)	34 (30–38)	33 (28–35)	34 (30–38)
**Diabetes treatment**					
• Diet	0/28 (0%)	1/19 (5.3%)	320/466 (68.7%)	0/3 (0%)	321/516 (62.2%)
• Metformin alone	0/28 (0%)	0/19 (0%)	8/466 (1.7%)	0/3 (0%)	8/516 (1.6%)
• Metformin with insulin	0/28 (0%)	14/19 (73.7%)	6/466 (1.3%)	0/3 (0%)	20/516 (3.9%)
• Insulin alone	28/28 (100%)	4/19 (21.1%)	132/466 (28.3%)	3/3 (100%)	167/516 (32.4%)
**Multiple pregnancy**	0/28 (0%)	3/19 (15.8%)	36/466 (7.7%)	0/3 (0%)	39/516 (7.6%)
**Smoking status**	4/28 (14.3%)	2/19 (10.5%)	68/466 (14.6%)	0/3 (0%)	74/516 (14.3%)
**BMI > 30 kg/m**^**2**^	6/28 (21.4%)	14/19 (73.7%)	238/466 (51.1%)	0/3 (0%)	258/516 (50%)
**Pregnancy-induced hypertension or pre-eclampsia**	12/28 (42.9%)	6/19 (31.6%)	64/466 (13.7%)	1/3 (33.3%)	83/516 (16.1%)
**Pre-existing hypertension**	3/28 (10.7%)	5/19 (26.3%)	23/466 (4.9%)	0/3 (0%)	31/516 (6%)
**Diabetic vascular disease**	14/28 (50%)	5/19 (26.3%)	0/466 (0%)	2/3 (66.6%)	21/516 (4.1%)
**History of perinatal death in previous pregnancy**	2/28 (7.1%)	4/19 (21.1%)	19/466 (4.1%)	0/3 (0%)	25/516 (4.8%)
**Assisted reproduction in this pregnancy**	5/28 (17.9%)	3/19 (15.8%)	70/466 (15%)	0/3 (0%)	78/516 (15.1%)
**Received antenatal steroids in this pregnancy**	11/28 (39.3%)	2/19 (10.5%)	86/466 (18.5%)	0/3 (0%)	99/516 (19.2%)

*T1DM*, type 1 diabetes mellitus; *T2DM*, type 2 diabetes mellitus; *GDM*, gestational diabetes mellitus; *IQR*, interquartile range; *BMI*, body mass index

## Discussion

### Key results

The purpose of this study was to describe how the type of maternal diabetes impacts neonatal outcomes. The admission rate was significantly higher in the T1DM cohort, as were the birth weight centiles and the rate of caesarean section. Of the NICU-admitted neonates, the T1DM cohort had the highest association of respiratory distress and the need for respiratory support. Relative to the T1DM and GDM cohorts, the T2DM group were most likely to be admitted to the NICU because of severe/refractory hypoglycaemia.

### Strengths and limitations

Strengths of the study included a large cohort of patients from a tertiary maternity hospital with both low- and high-risk pregnancies.

There are limitations to the conclusions we can reach due to the retrospective, single-centre study design. However, this represents a very large single cohort of neonates. Limitations include an unbalanced dataset, with the majority of neonates born to mothers with GDM. This is likely a natural consequence of the incidence of diabetes subtypes in women of childbearing age, with pregestational diabetes being rarer than GDM [19, 20]. Therefore, categorical data analysis was limited to norms of women with pregnancies complicated by GDM.

It was beyond the scope of this study to compare neonatal outcomes with neonates of mothers without diabetes who require NICU admission.

Additionally, maternal co-morbidities were not collected for non-admitted neonates, and factors such as maternal smoking, raised BMI, or pre-eclampsia possibly confounded the admission risk. However, it is worth noting that many of these co-morbidities are not normally distributed in the diabetes population secondary to microvascular disease.

### Interpretation

An estimated 16.9% of pregnancies are affected by hyperglycaemia worldwide [21], similar to our institution (15.8%). We found an increased NICU admission requirement in neonates of mothers with pregestational diabetes compared to those without, while the admission rate for infants of GDM pregnancies is similar to the background population. Newman et al. reported a higher admission rate in T1DM (52.7%) but similar in T2DM (32.8%) [7]. Watson et al. also demonstrated higher admission rates for the T2DM and GDM cohorts (40% and 29%). However, a Dutch post hoc randomised controlled trial analysis reported lower admission rates of 20 and 21% for T1DM and combined T2DM and GDM cohorts [22], as did an Indian retrospective study on T2DM (23.8%) [23]. Our findings provide valuable evidence-based local data for counselling expectant mothers.

Rademaker demonstrated lower rates of neonatal hypoglycaemia;T1DM (89.3% versus 57%) and combined T2DM and GDM (49.7% versus 32%) however, their cohort is not directly comparable, as it included non-admitted neonates and used a lower threshold of hypoglycaemia at 2.6 mmol/l [24]. Our hypoglycaemia rate, as a secondary complication amongst admitted neonates, was similar to that of Watson et al. in the GDM group (48.7% versus 49%) but higher in the T2DM cohort (73.7% versus 58%) [8]. However, our definition of hypoglycaemia used a higher blood glucose value (< 3.0 mmol/l versus 2.6 mmol/l), due to the system of measurement of point-of-care glucose using the Accu-Chek Inform II machine. A similar percentage of our neonates in the GDM group required support for respiratory distress (36.7% versus 38%) while fewer in our T2DM group needed respiratory support (15.8% versus 50%) [8].

Fewer neonates were admitted with severe or refractory hypoglycaemia as an initial indication than those who developed hypoglycaemia as a complication during admission (17.2% versus 51.6%). Robust guidelines for the management of hypoglycaemia on the postnatal wards and the introduction of 40% oral dextrose gel mean the majority of patients with a blood glucose < 3.0 mmol/l are not admitted to NICU as per local institutional policy ([24–27]).

### Generalisability

Within our cohort, neonates with T1DM were found to be on higher centiles for weight and gestational age, were born sooner, and were more likely to be delivered by caesarean section, all of which are associated with transient tachypnoea of the newborn. This is consistent with studies reporting an increase in large-for-gestational-age and caesarean section in T1DM cohorts [7, 22]. Increasing birth weight in T1DM pregnancies is multifactorial in aetiology. It can reflect maternal hyperglycaemia and fetal hyperinsulinemia, leading to macrosomia. Desoye et al. suggest two overgrowth phenotypes, independent of birth weight. Firstly, when mothers have near-normal pre- and peri-conceptual glycaemia, this improves micro-vascularisation of the developing placenta. If these mothers later develop hyperglycaemia, fetal overgrowth and a large-for-gestational-age neonate arise, due to unimpeded nutrient flow throughout pregnancy. On the other hand, impaired placentation due to maternal pre-conceptual hyperglycaemia may restrict fetal growth. If this is later counteracted by maternal hyperglycaemia and subsequent fetal overnutrition, it may result in a normal birth weight. Therefore, blood glucose levels in the antenatal and perinatal period determine fetal nutrition, growth, and birthweight [28].

In the context of T1DM, if macrosomia is suspected in the third trimester, iatrogenic delivery is intended primarily to prevent stillbirth. Elective caesarean section is frequently required because of unsuitability for induction of labour (IOL), in the context of an unfavourable cervix. Increased rates of caesarean section in those with diabetes in pregnancy occur for a multitude of reasons: macrosomia, fetal distress, cephalopelvic disproportion, and failed induction of labour [29, 30] but may also be influenced by physician decision-making and performance [31]. The general caesarean section rate for all pregnancies in our hospital is 34–37%, and 55% of NICU-admitted neonates born to mothers with diabetes were born via caesarean section. We did not record parity or history of previous sections, which may have influenced the rates in our cohort. Higher rates of co-morbidities such as pre-existing hypertension and pregnancy-induced hypertension/pre-eclampsia may have contributed to the increased caesarean section rate for maternal reasons in our pregestational diabetes mothers. While we did not collect HbA1 C data, T1DM mothers are likely to have longer diabetes duration and increased diabetes-related vascular diseases such as pregnancy-induced hypertension and pre-eclampsia. However, our pre-existing hypertension rates are notably higher in the T2DM cohort than previously reported (26.3% versus 16.4% [7]), possibly reflective of the higher proportion with BMI > 30 kg/m^2^ in our cohort (73.7% versus 51.6%) [7].

T1DM pregnancies showed an increased presence of meconium, a rate of emergency caesarean, and earlier admission to NICU, suggesting increased fetal distress around the time of delivery relative to the T2DM and GDM groups. The increased need for respiratory support and intravenous fluids, possibly due to hypoglycaemia, feeding intolerance, and earlier gestational age, may lead to the increased length of stay in T1DM neonates.

Our T2DM cohort were more likely to be admitted for severe/refractory hypoglycaemia relative to T1DM and GDM cohorts. However, hypoglycaemia subsequently evolved during the NICU stay for the majority of T1DM neonates. The high prevalence of respiratory morbidity observed in these neonates likely results in early recognition and treatment of hypoglycaemia in the NICU setting, before hypoglycaemia becomes severe. Due to neonates’ reduced ability to feed secondary to respiratory morbidity, intravenous dextrose fluids are commenced, preventing a severe drop in blood glucose. In contrast, T2DM and GDM groups have lower respiratory morbidity rates, and hypoglycaemia is often recognised on the postnatal ward, where infants may experience recurrent episodes of less severe hypoglycaemia (> 1.8 mmol/l) before requiring NICU admission. This reinforces the need for heightened vigilance for hypoglycaemia amongst neonates of pregestational diabetes pregnancies whose respiratory transition is satisfactory to avoid initial NICU admission. Our hospital recommends early feeding within the first hour of birth and pre-feed point-of-care glucose measurements using the Accu-Chek Inform II machine after the first feed. A blood glucose ≤ 1.8 mmol/l or persistent blood glucose ≤ 2.6 mmol/l despite two doses of 40% dextrose gel requires immediate NICU admission [14].

### Parental counselling

The admission of a newborn to the NICU is a potentially distressing time for parents. Effective parental counselling is paramount in managing anxieties and expectations. Language should be simple, avoiding medical jargon, and information should be consistent, delivered in bite-sized amounts and repeated often by the multi-disciplinary team [13]. It should be individualised to the parents’ needs, addressing their fears, expectations, and acceptance of their infants’ clinical condition [13]. Involving them in caregiving and decision-making should be encouraged [32]. Providing structured evidence-based counselling to parents could reduce stress and improve bonding. Factors such as parental education, culture, and socio-economic background, as well as previous neonatal death and fertility journey, should be taken into account [33].

### Research implications

We achieved our aim of describing how the type of maternal diabetes impacts NICU admissions, providing accurate contemporaneous, local data to support healthcare professionals in counselling patients with diabetes in pregnancy. A follow-up prospective analysis could assess the impact of confounding maternal factors on neonatal morbidity and NICU admission.

Future research would focus on gestation-specific neonatal outcomes in those born to mothers with pregestational and gestational diabetes. Despite a large cohort in our study, the small numbers born at each gestational week limit risk assessment by gestational age. Multiple tertiary centres may be required to achieve adequate patient numbers to explore this further.

## Conclusion

This study demonstrates that up to 41.8% of mothers with T1DM and a third of mothers with T2DM can expect to have neonates admitted to NICU, while GDM pregnancies have admission rates similar to the non-diabetes population. Mothers with pregestational diabetes were more likely to be discharged home while their infant remained in NICU. Within our cohort, neonates with maternal T1DM had higher birth weight centiles, were born earlier, and were more likely to be delivered by caesarean section. The T2DM cohort were most likely to be admitted with severe/refractory hypoglycaemia, possibly reflecting their otherwise uncomplicated neonatal course. They require early identification and monitoring of glucose levels after delivery. However, the overall incidence of hypoglycaemia was greatest in the T1DM cohort who were admitted with respiratory or other problems. This has important implications for antenatal counselling in preparing expectant parents for this eventuality, as well as developing key NICU services such as lactation support for separated maternal-infant dyads.

## Supplementary Information

Below is the link to the electronic supplementary material.

Supplementary file1 (DOCX 36 KB)

Supplementary file2 (DOCX 16 KB)

Supplementary file3 (DOCX 65 KB)

Supplementary file4 (DOCX 59 KB)

## Data Availability

No datasets were generated or analysed during the current study.

## Abbreviations

BMI: Body mass index
CDH: Congenital diaphragmatic hernia
CTG: Cardiotocography
GDM: Gestational diabetes mellitus
HbA1c: Glycated haemoglobin
HIE: Hypoxic ischaemic encephalopathy
HIPE: Hospital In-Patient Enquiry system
INSURE: Intubation-surfactant-extubation
IQR: Interquartile range
IUGR: Intrauterine growth restriction
LBW: Low birth weight
LSCS: Lower segment caesarean section
MODY: Mature onset diabetes of the young
NICU: Neonatal intensive care unit
NIV: Non-invasive ventilation
SVD: Spontaneous vaginal delivery
T1DM: Type 1 diabetes mellitus
T2DM: Type 2 diabetes mellitus
