# Supplementary material for: Increased risk of admission to neonatal intensive care unit in neonates born to mothers with pregestational diabetes
Source: Eur J Pediatr. 2025 May 22;184(6):354. doi: 10.1007/s00431-025-06170-0 (PMC12098415; doi:10.1007/s00431-025-06170-0)
Supplement: Supplementary file 1 — Supplementary file1 (DOCX 36 KB) [file 431_2025_6170_MOESM1_ESM.docx]

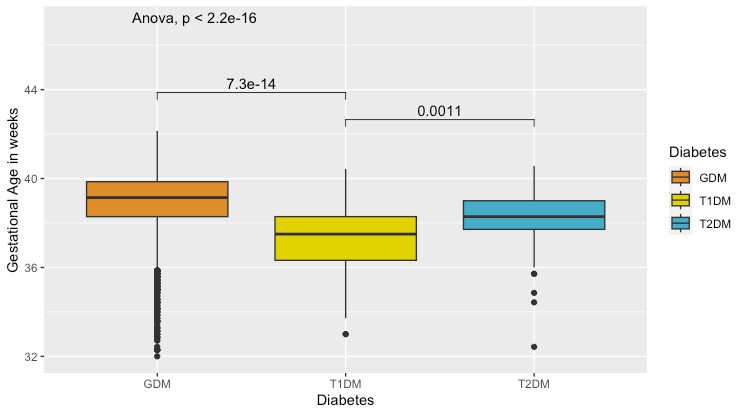


Appendix 1. Box plot comparing gestational age at birth amongst three cohorts of neonates born to mothers with pre-and gestational diabetes. T1DM, type 1 diabetes mellitus; T2DM, type 2 diabetes mellitus; GDM, gestational diabetes mellitus
