# Supplementary material for: Increased risk of admission to neonatal intensive care unit in neonates born to mothers with pregestational diabetes
Source: Eur J Pediatr. 2025 May 22;184(6):354. doi: 10.1007/s00431-025-06170-0 (PMC12098415; doi:10.1007/s00431-025-06170-0)
Supplement: Supplementary file 2 — Supplementary file2 (DOCX 16 KB) [file 431_2025_6170_MOESM2_ESM.docx]

# Appendix 2. Table demonstrating maternal obstetric features of neonates born to mothers with pre- and gestational diabetes who were admitted to NICU

| **Neonatal characteristics** | **Neonates born to mothers with**  **T1DM**  **(n=28)** | **Neonates born to mothers with T2DM**  **(n=20)** | **Neonates born to mothers with GDM**  **(n=469)** | **Neonates born to mothers with MODY (n = 3)** | **All neonates born to mothers with pre – and gestational diabetes**  **(n=520)** |
| --- | --- | --- | --- | --- | --- |
| **Mode of delivery**   - **SVD** - **Instrumental** - **Elective LSCS** - **Emergency LSCS** | 3/28 (10.7%)  1/28 (3.5%)  12/28(42.9%)  12/28 (42.9%) | 7/20 (35%)  2/20 (10%)  7/20 (35%)  4/20 (20%) | 148/469(31.6%)  69/469 (14.7%)  125/469(26.7%)  127/469(27.1%) | 2/3 (66.7%)  1/3 (33.3%)  0/3 (0%)  0/3 (0%) | 160/520(30.8%)  73/520 (14%)  144/520(27.7%)  143/520(27.5%) |
| **Induction of labour** | 8/28 (14.3%) | 8/20 (40%) | 188/469 (40.1%) | 3/3 (100%) | 207/520 (39.8%) |
| **Meconium liquor** | 3/28 (10.7%) | 2/20 (10%) | 56/469 (11.9%) | 0/3 (0%) | 61/520 (11.7%) |
| **Pathological CTG** | 6/28 (21.4%) | 5/20 (25%) | 148/469 (31.6%) | 0/3 (0%) | 159/520 (30.5%) |

T1DM, type 1 diabetes mellitus; T2DM, type 2 diabetes mellitus; GDM, gestational diabetes mellitus; MODY, mature onset diabetes; SVD, spontaneous vaginal delivery; LSCS, lower segment caesarean section; CTG, cardiotocography
