# Supplementary material for: Increased risk of admission to neonatal intensive care unit in neonates born to mothers with pregestational diabetes
Source: Eur J Pediatr. 2025 May 22;184(6):354. doi: 10.1007/s00431-025-06170-0 (PMC12098415; doi:10.1007/s00431-025-06170-0)
Supplement: Supplementary file 3 — Supplementary file3 (DOCX 65 KB) [file 431_2025_6170_MOESM3_ESM.docx]

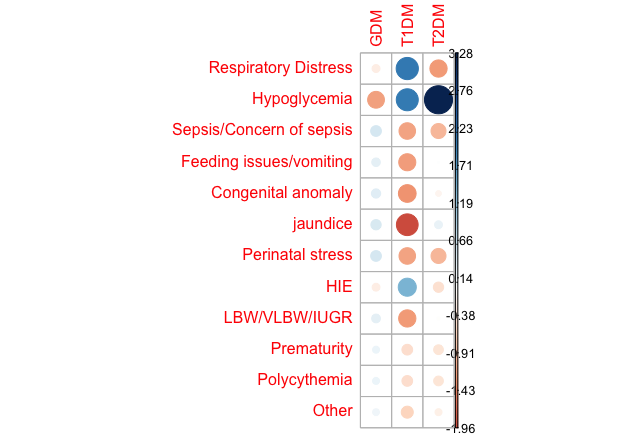


Appendix 3. Correlation plot demonstrating relative proportions of admissions by cause across three cohorts of neonates born to mothers with pre-existing and gestational diabetes. T1DM, type 1 diabetes mellitus; T2DM, type 2 diabetes mellitus; GDM, gestational diabetes mellitus; HIE, hypoxic-ischaemia encephalopathy; LBW, low birth weight; VLBW, very low birth weight; IUGR, intrauterine growth restriction. Blue represents positive correlations and red represents negative correlations.  x^2^ = 39.142, degrees of freedom=22 and p=0.0139.
