# Supplementary material for: Increased risk of admission to neonatal intensive care unit in neonates born to mothers with pregestational diabetes
Source: Eur J Pediatr. 2025 May 22;184(6):354. doi: 10.1007/s00431-025-06170-0 (PMC12098415; doi:10.1007/s00431-025-06170-0)
Supplement: Supplementary file 4 — Supplementary file4 (DOCX 59 KB) [file 431_2025_6170_MOESM4_ESM.docx]

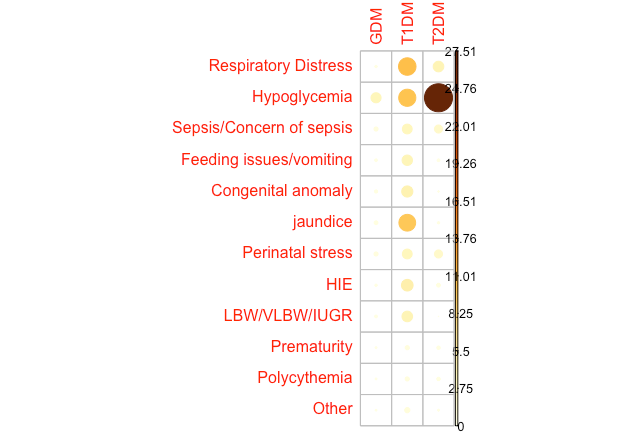


Appendix 4. Correlation plot of demonstrating relative contribution of reason for admission across three cohorts of neonates born to mothers with pre-existing and gestational diabetes. T1DM, type 1 diabetes mellitus; T2DM, type 2 diabetes mellitus; GDM, gestational diabetes mellitus; HIE, hypoxic-ischaemia encephalopathy; LBW, low birth weight; VLBW, very low birth weight; IUGR, intrauterine growth restriction. The size and shade of correlation represents the degree of relative contribution (darker/larger circles represent a greater degree than expected admissions)
